# Death by Starvation

**Published:** 1900-08-11

**Authors:** 


### DEATH BY STARVATION.

Dr. Vivian Poore[1] says that cases of death from starvation may be divided into two classes. First of all, cases in which solid food is withheld and water is obtainable; and next, cases where both food and drink are withheld—that is to say, in which death occurs from hunger and thirst combined. Death from the latter cause is very much more rapid than in cases where food alone is withheld. Death where water is withheld is probably death from uræmia, because the body is not flushed, and the waste products are not carried away by the kidneys; and the person generally dies delirious and often comatose, the body smelling very foul. Death from lack of food only is a totally different thing, for whereas a person will only live a week at the outside where both food and water are withheld, we know as a result of music-hall experiments that a man may go on living for six weeks with no food, or with very little food indeed. There may, perhaps, have been trickery in some of these experiments. A man may get a good deal of food in the guise of water; he may get colourless alcoholic food, and syrups and albumen; and thus where water is given *ad libitum* a good deal of food may be incorporated with it. Still, these exhibitions have not been all trickery. It is certain that those people who have exhibited themselves for money and fasted for six weeks have lost a prodigious amount of weight, that is to say several stones, and from the experience so gained we may learn this useful lesson, that if people are put in circumstances where although no food is obtainable water can be had, they may know that if they keep quiet they have a reasonable chance of escape. Take, for instance, entombed miners. In a mine there may be water but no food, and if it is known that by taking water but no food, and keeping quiet, one may survive until rescued, it is a very important fact. We may add that the knowledge that life can be maintained for a very considerable time without any food but water might well be utilised more than is sometimes done in the management of certain surgical cases in which it is necessary to ensure quietude of the stomach for some time after operation. The hardship and the misery caused by lack of food are felt with the greatest keenness during the first few days, and it is by no means clear that it would not be well to get over this difficulty first by a preliminary training in starvation.

[1] Clinical Jour., July 4.

